# Impact of continuous home care on quality of life and depression status in patients with acute coronary syndrome

**DOI:** 10.3389/fcvm.2026.1700557

**Published:** 2026-02-02

**Authors:** Hong Li, Yan Wang, Yaqun Yu

**Affiliations:** 1 Department of Cardiovascular Medicine, Suzhou Research Center of Medical School, Suzhou Hospital, Affiliated Hospital of Medical School, Nanjing University, Suzhou, China; 2 Department of Nursing, Shanghai Municipal Hospital of Traditional Chinese Medicine, Shanghai University of Traditional Chinese Medicine, Shanghai, China; 3 Department of Cardiac Vascular Surgery, The First Medical Center of the People’s Liberation Army General Hospital, Beijing, China

**Keywords:** acute coronary syndrome, continuous home care, depression, prognosis, quality of life

## Abstract

**Objective:**

Our aim in this study is to evaluate the impact of continuous home-based care on depressive symptoms, quality of life, and major adverse cardiovascular events in patients with acute coronary syndrome (ACS) and mild-to-moderate depression.

**Methods:**

A total of 200 patients with ACS diagnosed in our hospital during the period between October 2021 and April 2024 were selected for the study. All patients were screened for depression using the PHQ-9 questionnaire 1–8 weeks after myocardial infarction. Patients with scores consistent with mild to moderate depression were included in the study. After baseline assessment, eligible participants were randomly assigned 1:1 to the routine care group or the continuous home care group using a computer-generated block-randomization sequence, stratified by depression severity and ACS type. Depression, quality of life, and adverse cardiovascular events in the two groups of patients at different time points were analyzed.

**Results:**

There were no statistical differences in clinical data and background between the two groups of patients included in the study (*P* > 0.05). However, as the follow-up time prolonged, the anxiety and depression scores of patients in the continuous home care group were significantly lower than those in the routine care group (EQOL-VAS score at 6 months’ follow-up: routine care group 70.31 ± 7.77, continuous home care group 79.69 ± 6.07, *P* = 0.043; EQOL-VAS score at 12 months’ follow-up: routine care group 63.82 ± 6.73, continuous home care group 74.42 ± 7.24, *P* = 0.046; EQOL-VAS score at 18 months’ follow-up: routine care group 54.15 ± 13.30, continuous home care group 63.94 ± 11.28, *P* = 0.038). In addition, compared with the routine care group, the quality of life of patients in the continuous home care group significantly improved, and the overall incidence of adverse cardiovascular events was lower (*P* < 0.05) in this group than in the former group.

**Conclusions:**

The results of the study showed that continuous home care can alleviate the anxiety and depression of patients with ACS, enhance their quality of life, reduce the incidence of adverse cardiovascular events, and improve their prognosis. After adjusting for confounding factors such as age, history of MI, heart failure, and medication adherence, the intervention effect did not show significant attenuation, further confirming its independent protective role.

## Introduction

Acute coronary syndrome (ACS) is a major type of mortality in individuals with cardiovascular disease (1, 2). Its serious condition, poor prognosis, and heavy economic burden have a huge impact on the physical and mental health of patients and their families, as well as the healthcare system (3, 4). ACS includes ST elevation myocardial infarction (STEMI), non-ST elevation myocardial infarction (NSTEMI), and unstable angina (UA), which are common syndromes leading to acute myocardial ischemia. The prevalence of ACS continues to increase, with an estimated 7 million new cases diagnosed worldwide every year (1).

The pathogenesis of ACS primarily involves the rupture or erosion of unstable atherosclerotic plaques in the coronary artery, followed by the formation of fresh thrombus, which eventually leads to acute cardiac ischemia (5). Cardiovascular disease is the leading cause of death among the Chinese population, with ACS being the primary type responsible for these deaths (6). Continuous care, a patient-centered model bridging hospital and community settings, is critical for ACS management. It involves structured, longitudinal support provided by multidisciplinary teams through regular follow-ups, education, and personalized interventions (7). Continuous home-based care shares the longitudinal philosophy of transitional care, which has been employed for decades to bridge hospital-to-community gaps and reduce rehospitalization rates. While transitional care typically focuses on the first 30–90 days postdischarge, our intervention extends structured multidisciplinary follow-up for 18 months and explicitly integrates depression management, thus building upon and expanding the earlier transitional-care framework. By addressing both medical and psychosocial needs of patients, this approach aims to enhance treatment adherence, prevent complications, and mitigate post-ACS depression (8). The management of ACS emphasizes reducing the occurrence of adverse cardiac events, improving the health status of patients, and optimizing their quality of life (9).

With the development of the “bio-psycho-social” medical model (10) and the introduction of the “dual-heart medicine” concept (11), the traditional thinking that the pathogenesis of coronary heart disease is limited to biological factors has been broken. The bio-psycho-social model posits that health outcomes are shaped by interactions among biological, psychological, and social determinants, while dual-heart medicine explicitly addresses the bidirectional relationship between cardiovascular diseases and mental health disorders (e.g., depression and anxiety). Studies have shown that anxiety and depression are closely related to the occurrence and development of coronary heart disease (12, 13). On the one hand, anxiety and depression are independent risk factors for coronary heart disease; on the other hand, the incurable nature, lifelong treatment, risk, and recurrent episodes of coronary heart disease impose severe physical, mental, economic, and social burdens on patients, which can further trigger or aggravate their emotional disorders (14). In addition, compared with patients with chronic coronary artery disease, ACS often causes more severe chest pain symptoms and requires more intensive medication treatment, including concerns about the uncertainties of interventional procedures, all of which make patients more susceptible to anxiety and depression (14). However, persistent anxiety and depression can significantly negatively impact the disease progression and prognosis of ACS patients, notably increasing their mortality (15).

The research results show that continuous care is negatively correlated with the severity of depression and anxiety disorders in patients with coronary heart disease. Improving social support and patients’ utilization of social support can reduce the severity of depression and anxiety and improve patients’ quality of life. In the context of ACS, social support encompasses emotional support (such as reassurance from family or nurses), informational support (such as disease education), and instrumental support (such as assistance with daily activities) (16). Continuous care models, such as the home-based intervention in this study, are designed to provide these multidimensional supports through structured follow-up (such as regular home visits, telehealth counseling, and community-based rehabilitation programs) (17). However, there is currently a lack of supporting evidence, which prompted this prospective cohort study to evaluate the impact of continuous home-based care on depressive symptoms, quality of life, and major adverse cardiovascular events (MACE) in patients with ACS and mild-to-moderate depression.

The aim of this study was to: (1) quantify the effects of continuous home care on depression and quality of life using validated scales (PHQ-9, EQOL-VAS); (2) determine whether psychosocial improvements translate into reduced cardiovascular events (e.g., ACS recurrence and stroke); and (3) provide a replicable model for integrating biopsychosocial support into routine post-ACS care. Findings will inform clinical guidelines on holistic ACS management and highlight the cost-effectiveness of community-based interventions.

## Methods

### Participants

A prospective cohort design was used in this study, which included patients diagnosed with ACS (including ST-elevation myocardial infarction, NSTEMI, and UA) during the period between October 2021 and April 2024. Potential participants were identified through daily screening of the electronic health records (EHRs) of our cardiology department. Allocation concealment to reduce the risk of bias was implemented using opaque envelopes, and blinding was set for outcome assessors. Given that the intervention itself could not be blinded, the possibility of implementation bias arose. To minimize this possibility, we conducted standardized personnel training and developed a prespecified analysis plan. All patients were screened for depression using the PHQ-9 questionnaire 1–8 weeks after myocardial infarction, and patients with scores consistent with mild to moderate depression were included in the study. The reason for selecting 1–8 weeks was that the American Heart Association (AHA) recommends screening for depression within 2–3 months of patients developing ACS, as symptoms may not be apparent during the initial hospitalization phase (18). In addition, mild to moderate depression (PHQ-9 ≥5) lasting more than 1 week is less likely to resolve spontaneously, ensuring that the included patients have clinically significant depressive symptoms (19). Exclusion criteria were as follows: (1) Severe cognitive impairment or inability to complete questionnaires. (2) Terminal illness (e.g., metastatic cancer) with life expectancy <6 months. (3) Participation in other cardiac rehabilitation trials. (4) History of severe mental illness (e.g., schizophrenia) unrelated to ACS.

The general clinical data of all selected patients, including gender, age, height, weight, smoking history, drinking history, history of combined medication, and past medical history, were collected. The nursing period lasted for 6 months and then the patients were followed up for 1 year; data on adverse cardiovascular events during the follow-up period were collected. The patients' depression states were evaluated using the PHQ-9 scale. The EuroQol-VAS scale and EuroQol-5D scale were used to evaluate the patients' quality of life. The ethics committee of our hospital approved this study, and all patients participating in the study signed the informed consent form.

### Patient grouping and care

According to different nursing methods, the patients were divided into two groups: the routine care group and the continuous home care group. After baseline assessments, eligible participants were randomly assigned 1:1 to the routine care group or continuous home care group using a computer-generated block randomization sequence (block size=4), stratified by depression severity (PHQ-9: 5–9 vs.10–14) and ACS type (STEMI/NSTEMI/UA). The allocation sequence was concealed in opaque envelopes opened by an independent research coordinator after enrollment.

Routine care group: (1) Psychological counseling: Nursing staff should regularly communicate with patients and observe their facial expressions and speech status to discern and understand any negative emotions. If they exhibit obvious negative or pessimistic emotions, the nursing staff should actively encourage them and remind their family members to comfort them. (2) Health education: The staff should remind the patients to actively correct their diet postsurgery, quit smoking and alcohol, and encourage them to undergo traditional Chinese medicine adjuvant therapy. Then, they should advise the patients’ family members to provide dietary and exercise guidance and offer thoughtful care to the patients. (3) Medication guidance: The nursing staff should confer with clinical doctors to provide routine medication treatment to the patients, explain the role and necessity of each medication to them, and ensure that the patients understand the importance of the medication treatment, accept them, and actively cooperate with the staff. (4) Discharge guidance: Before discharge, the staff should explain the contents of the doctors’ orders to the patients in detail and remind them to pay attention to follow-up visits.

Continuous home care group: All patients in this group received continuous home care services, as follows. (1) Formation of a service team: All cardiology nurses formed a service team, called “Continuous Service Group.” Every 2–3 nursing staff formed a team freely, and there were four teams in total. The head nurse was the team leader and assigned tasks. The four teams were responsible for network services, telephone services, community services, and home services, respectively. (a) Network services: Services were provided to patients through online platforms such as WeChat, QQ, and email, including educating the patients and their families about myocardial infarction, the importance of understanding the condition, and preventing recurrence. The patients were trained on how to self-monitor vital signs and recognize warning signals. (b) Telephone services: Telephone services were provided to the patients every Friday morning, and the patients and their families were contacted to ascertain the patients’ recovery status, inquire about any discomfort experienced by them, and provide appropriate guidance. The patients were reminded to seek follow-up care if any abnormalities were detected. (c) Community services: After discharge, the patients registered at their community hospital, where healthcare providers fully understood their hospital treatment and medication history. A comprehensive community rehabilitation and home care plan for the patients was established, including the creation of a community medical record. This record documented the patients’ medication treatment, daily living habits, diet, exercise, and physical conditions. Regular first aid training was provided, teaching family members how to respond to acute myocardial infarction suffered by the patients, including their level of familiarity with cardiopulmonary resuscitation (CPR) and the use of automatic external defibrillator (AED). (d) Home service: Monthly home visits were conducted by nurses to assess health behavior compliance, physical recovery, and emotional state. If severe negative emotions were identified, they were addressed promptly. Nurses monitored changes in the patients’ condition and documented these for physician reference. In addition, the nursing team ensured active communication among the team members, regularly updating the patients’ health status and care plan.

### Anxiety and depression evaluation

The anxiety and depression status of the patients were evaluated by using the PHQ-9 scale, the Montgomery–Asberg Depression Rating Scale (MADRS), and the Cardiac Anxiety Questionnaire (CAQ). The mental status of the patients was evaluated at baseline, 6, 12, and 18 months.

The PHQ-9 scale is a brief self-assessment questionnaire for depression. Related studies have confirmed that it has high accuracy for depression screening (20). The questionnaire contains nine items, each corresponding to four different levels of options, with the following scoring: 0 = never, 1 = several days, 2 = often (more than half of the time in the past 2 weeks), and 3 = nearly every day. If the total score is greater than 10 points, it can be defined as “severe depression.”

The MADRS is a 10-item scale developed by Montgomery of Guy's Hospital in London and Asberg from the Stockholm Institute (21). It is used to reflect the effect of antidepressant treatment and monitor the patient's condition. A score of 0–6 indicates normal or asymptomatic, 7–19 indicates mild depression, 20–34 indicates moderate depression, and more than 34 indicates severe depression.

The CAQ is a scale developed by the Department of Psychology at West Virginia University in the United States to evaluate the severity of cardiovascular-related anxiety symptoms. It includes three dimensions: fear and anxiety, avoidance, and excessive attention to heart conditions. Each dimension contains 5–8 items, totaling 18 items. It is a self-assessment five-point scale, in which symptoms are rated on a scale of five levels: “never” is 0 points, “rarely” is 1 point, “sometimes” is 2 points, “often” is 3 points, and “always” is 4 points. Higher scores indicate more severe cardiovascular-related anxiety, with a total score exceeding 30 points considered positive for significant psychological distress (22).

### Quality-of-life evaluation

The European Quality of Life Five Dimensions (EQOL-5D) scale was designed by the European Quality of Life Association and can be used for both general and specific population health evaluations (23). The complete EQOL-5D scale consists of two parts: the first part contains three modules (five-dimensional measurement, intuitive health scale, and respondent basic information scale), and the second part contains an assessment questionnaire for specific health conditions. The first part contains five dimensions: mobility, self-care, usual activities, pain or discomfort, and anxiety or depression. Each dimension contains three levels: no problems, some problems, and unable. The scoring rule is based on the EQVAS, which is a 20 cm vertical visual analog scale, in which the top end represents a score of 100, indicating “the best health status in mind,” and the bottom end represents a score of 0, indicating “the worst health status in mind.”

### Statistical analyses

Statistical analyses were performed using SPSS 25.0 software. A single-sample normal distribution test was performed on each quantitative data. Data that followed a normal distribution were presented as mean ± standard deviation, and a *t*-test was used for intergroup comparison. Data that did not follow a normal distribution were presented as median (P25, P75), and a non-parametric test was used for intergroup comparison. Count data were expressed as case number and/or percentage, and a chi-square test was used for comparison. Correlation analyses between depression scores (PHQ-9, MADRS, and CAQ) and QOL (EQOL-VAS) were performed at each assessment time point (baseline, 6, 12, and 18 months) using Pearson's/Spearman's tests. Longitudinal changes in these relationships were explored using mixed-effects models. A generalized linear mixed model (GLMM) was used to adjust for potential confounding factors such as baseline age, gender, hypertension, diabetes, previous MI, left ventricular ejection fraction (LVEF), and medication adherence. The intervention group was treated as a fixed effect, while individuals were considered random effects. A value of *P* < 0.05 was considered statistically significant.

## Results

### Comparison of clinical characteristics

**Figure 1:**
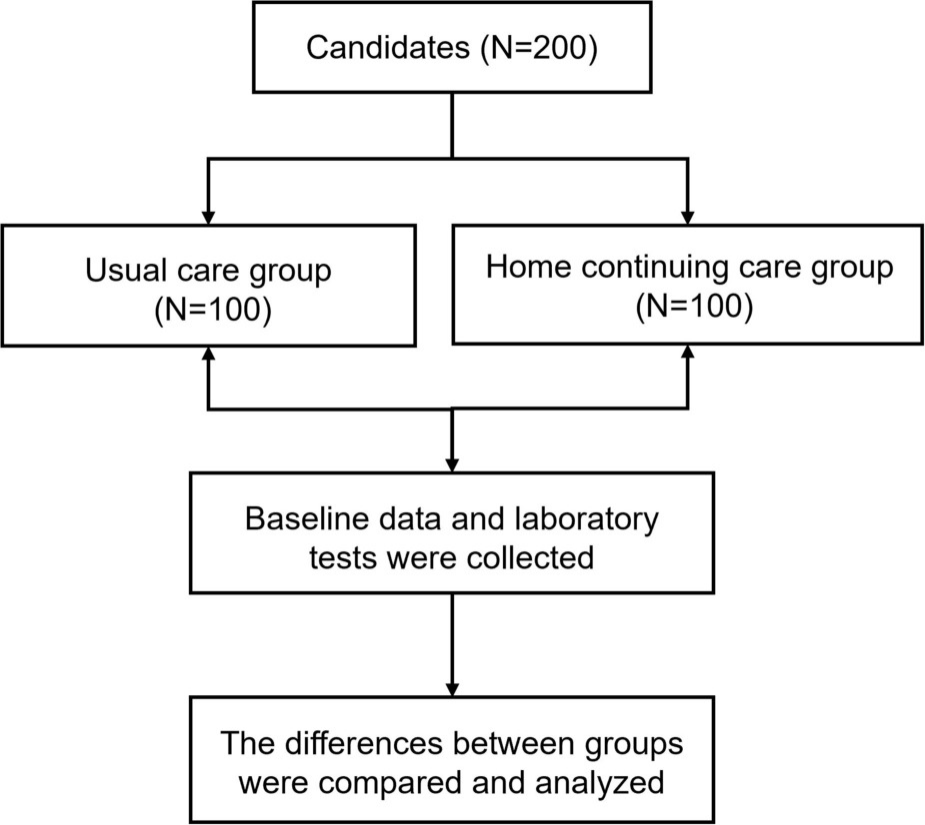
Enrollment of the study participants in the primary cohort.

Of the 1,203 ACS patients screened between October 2021 and April 2024, 320 met initial eligibility criteria. After a PHQ-9 assessment, 200 patients (62.5%) with scores ≥5 were randomized (100 per group). During follow-up, five deaths occurred in the routine care group and four patients were lost in the home care group, leaving 95 and 96 patients for the final analysis, respectively (Figure 1). Allocation was solely based on randomization, with no influence from patient preferences or clinician judgment. As shown in Table 1, the two groups were well-balanced in terms of demographics, medical history, and baseline depression scores (all *P* > 0.05), confirming successful randomization.

**Table 1:** Clinical characteristics of the routine care group and continuous home care group.

Characteristic	Routine care group (*N* = 100)	Continuous home care group (*N* = 100)	*P*-value
Male, *n* (%)	44 (44)	36 (36)	0.248
Age (years)	67.60 ± 6.90	70.64 ± 6.75	0.562
BMI (kg/ m^2^)	24.05 ± 2.89	23.16 ± 2.82	0.601
Smoker, *n* (%)	28 (28)	32 (32)	0.537
Drinking, *n* (%)	27 (27)	29 (29)	0.753
Education, *n* (%)			0.888
≤High school	50 (50)	49 (49)	
≥College	50 (50)	51 (51)	
Lives alone, *n* (%)	16 (16)	21 (21)	0.363
Occupation, *n* (%)			0.836
Employed	56 (56)	58 (58)	
Unemployed	4 (4)	2 (2)	
Retired	37 (37)	36 (36)	
Sick leave	2 (2)	3 (3)	
Other	1 (1)	0 (0)	
Taking psychotropic medicine, *n* (%)			0.858
Yes	19 (19)	20 (20)	
No	81 (81)	80 (80)	
Medical history, *n* (%)			
Myocardial infarction	19 (19)	17 (17)	0.713
Diabetes	21 (21)	19 (19)	0.724
Hypertension	38 (38)	41 (41)	0.664
Hyperlipidemia	16 (16)	17 (17)	0.849
Stroke	2 (2)	4 (4)	0.407
Heart failure	4 (4)	2 (2)	0.407
Medical therapy, *n* (%)			
Aspirin	99 (99)	98 (98)	0.561
P2Y12 inhibitor	100 (100)	100 (100)	-
Beta-blocker	69 (69)	68 (68)	0.879
Angiotensin converting enzyme inhibitor	70 (70)	72 (72)	0.755

### Anxiety and depression status in both groups

**Figure 2:**
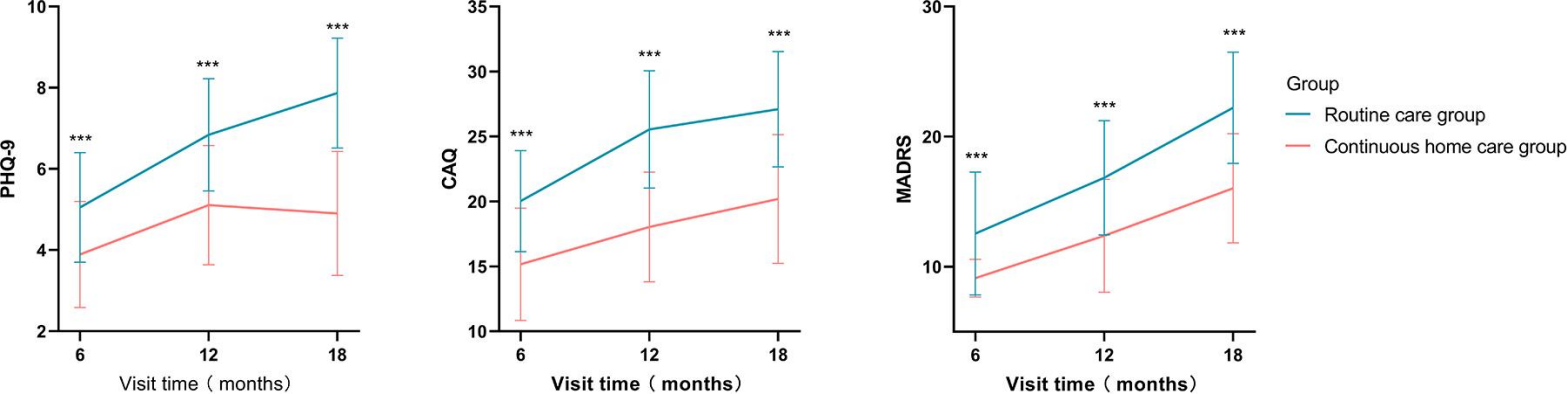
Differences in anxiety and depression scores between the continuous home care group and the routine care group (*P < 0.05, **P < 0.01, ***P < 0.001).

As shown in Figure 2, the study collected anxiety and depression indicators PHQ-9, CAQ, and MADRS scores of the two groups of patients at 6, 12, and 18 months. The results found that at 6 months’ follow-up, the PHQ-9 score was 5.05 ± 1.35 in the routine care group and 3.89 ± 1.3 in the continuous home healthcare group (*P* < 0.001); at 12 months’ follow-up, the PHQ-9 score was 63.82 ± 6.73 in the routine care group and 74.42 ± 7.24 in the continuous home healthcare group (*P* < 0.001); at 18 months’ follow-up, the PHQ-9 score was 54.15 ± 13.30 in the routine care group and 63.94 ± 11.28 in the continuous home healthcare group (*P* < 0.001).

At 6 months’ follow-up, the CAQ score was 20.03 ± 3.89 in the routine care group and 15.17 ± 4.32 in the continuous home healthcare group (*P* < 0.001); at 12 months’ follow-up, the CAQ score was 25.55 ± 4.51 in the routine care group and 18.03 ± 4.22 in the continuous home healthcare group (*P* < 0.001); at 18 months’ follow-up, the CAQ score was 54.15 ± 13.30 in the routine care group and 20.19 ± 4.94 in the continuous home healthcare group (*P* < 0.001).

At 6 months’ follow-up, the MADRS score was 12.55 ± 4.73 in the routine care group and 9.12 ± 1.44 in the continuous home healthcare group (*P* < 0.001); at 12 months’ follow-up, the MADRS score was 16.84 ± 4.39 in the routine care group and 12.38 ± 4.33 in the continuous home healthcare group (*P* < 0.001); at 18 months’ follow-up, the MADRS score was 22.22 ± 4.28 in the routine care group and 16.02 ± 4.21 in the continuous home healthcare group (*P* < 0.001) (Table 2). The study found that both depression and anxiety scores of patients in the continuous home care group were lower than those in the routine care group.

**Table 2:** Evaluation of quality of life during follow-up in the continuous home care group and the routine care group.

Characteristic	6-month visit			12-month visit			18-month visit		
	Routine care group (*N* = 100)	Continuous home care group (*N* = 100)	*P*	Routine care group (*N* = 99)	Continuous home care group (*N* = 98)	*P*	Routine care group (*N* = 95)	Continuous home care group (*N* = 96)	*P*
EuroQol-VAS, points	70.31 ± 7.77	79.69 ± 6.07	0.043	63.82 ± 6.73	74.42 ± 7.24	0.046	54.15 ± 13.30	63.94 ± 11.28	0.038
EuroQol-5 domains, *n* (%)									
Pain/discomfort			0.015			0.016			0.044
No	73 (73%)	89 (89%)		59 (59.59%)	77 (78.57%)		60 (63.15%)	73 (76.04%)	
Moderate	24 (24%)	9 (9%)		35 (35.35%)	18 (18.36%)		26 (27.36%)	21 (21.87%)	
Extreme	3 (3%)	3 (3%)		5 (5.05%)	3 (3.06%)		9 (9.47%)	2 (2.08%)	
Anxiety/Depression			0.015			0.006			0.005
No	63 (63%)	78 (78%)		57 (57.57%)	77 (78.57%)		48 (50.52%)	70 (72.91%)	
Moderately	32 (32%)	22 (22%)		31 (31.31%)	18 (18.36%)		34 (35.78%)	21 (21.8%)	
Extremely	5 (5%)	0		10 (10.10%)	3 (3.06%)		13 (13.68%)	5 (5.20%)	
Mobility			<0.001			0.005			0.033
No problems	68 (68%)	89 (89%)		64 (64.64%)	83 (84.69%)		55 (57.8%)	71 (73.9%)	
Some problems	32 (32%)	11 (11%)		34 (34.34%)	14 (14.28%)		37 (38.9%)	22 (22.91%)	
Confined to bed	0	0		1 (1.01%)	1 (1.02%)		3 (3.15%)	1 (1.04%)	
Self-care			0.023			0.025			0.019
No problems	83 (83%)	95 (95%)		79 (79.79%)	91 (92.85%)		71 (74.73%)	86 (89.58%)	
Some problems	16 (16%)	5 (5%)		19 (19.19%)	7 (7.14%)		16 (16.84%)	5 (5.20%)	
Unable	1 (1%)	0		1 (1.01%)	0		8 (8.42%)	5 (5.20%)	
Usual activities			0.007			0.035			0.007
No problems	70 (70%)	88 (88%)		72 (72.72%)	85 (88.77%)		51 (53.6%)	71 (73.95%)	
Some problems	27 (27%)	11 (11%)		22 (22.22%)	12 (12.24%)		38 (40%)	24 (25%)	
Unable	3 (3%)	1 (1%)		5 (5.05%)	1 (1.02%)		6 (6.31%)	1 (1.04%)	
PHQ-9 score	5.05 ± 1.35	3.89 ± 1.3	*P* < 0.001	6.84 ± 1.38	5.11 ± 1.46	*P* < 0.001	7.87 ± 1.35	4.90 ± 1.52	*P* < 0.001
CAQ score	20.03 ± 3.89	15.17 ± 4.32	*P* < 0.001	25.55 ± 4.51	18.03 ± 4.22	*P* < 0.001	27.11 ± 4.44	20.19 ± 4.94	*P* < 0.001
MADRS score	12.55 ± 4.73	9.12 ± 1.44	*P* < 0.001	16.84 ± 4.39	12.38 ± 4.33	*P* < 0.001	22.22 ± 4.28	16.02 ± 4.21	*P* < 0.001

### Quality of life in both groups

The study analyzed changes in the quality of life (QOL) of the two groups during follow-up. The results showed that the QOL scores (EQOL-VAS and EQOL-5D) in the continuous home care group were significantly higher than those in the routine care group (*P* < 0.05). Whether at 6, 12, or 18 months of follow-up, the quality of life of patients in the continuous home care group was better than that in the routine care group (6-month follow-up EQOL-VAS score: routine care group 70.31 ± 7.77, continuity home care group 79.69 ± 6.07, *P* = 0.043; 12-month follow-up EQOL-VAS score: routine care group 63.82 ± 6.73, continuity home care group 74.42 ± 7.24, *P* = 0.046; 18-month follow-up EQOL-VAS score: routine care group 54.15 ± 13.30, continuity home care group 63.94 ± 11.28, *P* = 0.038). In the five dimensions of pain, anxiety/depression, mobility, self-care, and usual activities, the home care group had a significantly higher proportion of “no problem” at each time point compared with the routine care group (all *P* < 0.05). The number of cases with severe problems also remained lower in the home care group than in the routine care group (all *P* < 0.05). All these findings indicated that continuous home care significantly improved the subjective health perception and multidimensional quality of life of patients with ACS within 18 months. The specific results are given in Table 2.

### Correlation analysis between quality of life and depression scores

As given in Table 3, the correlation between the PHQ-9 and the CAQ depression symptom scores at 6 months’ follow-up and the EQOL-VAS was not significant (*P* = 0.127, *P* = 0.255). At the 6-month follow-up, the MADRS scores were significantly negatively correlated with EQOL-VAS, with a correlation coefficient of *r* = −0.251 and *P* < 0.001. At the 12- and 18-month follow-ups, depression symptom scores (PHQ-9, MADRS, and CAQ) were significantly negatively correlated with quality of life (EQOL-VAS) at all follow-up visits (all *P* < 0.05). Among these, the correlation between PHQ-9 and EQOL-VAS at the 18-month follow-up was the strongest, with a correlation coefficient of −0.435 and *P* < 0.001.

**Table 3:** Correlation between quality of life and depression scores.

Characteristic	EuroQol-VAS-6		EuroQol-VAS-12		EuroQol-VAS-18	
	*r*	*P*	*r*	*P*	*r*	*P*
PHQ-9 score	−0.108	0.127	−0.255	<0.001	−0.435	<0.001
CAQ score	−0.081	0.255	−0.353	<0.001	−0.342	<0.001
MARDS score	−0.251	<0.001	−0.216	0.002	−0.286	<0.001

### Adverse cardiovascular events

The study also analyzed the adverse cardiovascular events of the two groups of patients during the follow-up period. The results showed that the nursing status affected the occurrence of adverse events in patients. The overall incidence of adverse events in the continuous home care group was significantly lower than that in the routine care group during the follow-up period (*P* < 0.05). Among them, five patients died of cardiovascular events in the routine care group, but none died in the continuous home care group (*P* = 0.024); ACS occurred in 28 patients in the routine care group and 14 patients in the continuous home healthcare group (*P* = 0.015); 9 patients in the routine care group and two patients in the continuous home care group had stroke (*P* = 0.030); 11 patients in the routine care group and three patients in the continuous home care group developed heart failure (*P* = 0.027); 24 patients received stent treatment in the routine care group and 12 patients received the treatment in the continuous home care group (*P* = 0.027). The detailed results are presented in Table 4.

**Table 4:** Adverse cardiovascular events during follow-up between the continuous home care group and the routine care group.

Characteristic	Routine care group (*N* = 100)	Continuous home care group (*N* = 100)	*P*
Cardiovascular-related death	5	0	0.024
ACS	28	14	0.015
Stroke	9	2	0.030
Revascularization	24	12	0.027
Heart failure	11	3	0.027

After a multivariate generalized linear mixed model adjustment for baseline age, gender, body mass index (BMI), hypertension, diabetes, previous myocardial infarction, heart failure, stroke, hyperlipidemia, and Morisky Medication Adherence Scale score, the continuous home care group still showed significant advantages: the average reduction in the CAQ score was 6.90 points (*P* < 0.001), the EQ-VAS quality-of-life score increased by 8.30 points (*P* < 0.001), and the risk of major adverse cardiovascular events decreased by 53% (OR 0.47, *P* = 0.006) (Table 5), confirming that the intervention effects were independent of the above confounding factors.

**Table 5:** Adjusted estimates of the intervention effects.

Outcome variable	*β*/OR	95% CI	*P*
PHQ−9	*β* = –1.72	–2.54 to –0.90	<0.001
MADRS	*β* = –5.30	–7.10 to –3.50	<0.001
CAQ	*β* = –6.90	–9.40 to –4.40	<0.001
EQ-VAS	*β* = 8.30	4.70–11.90	<0.001
MACE	OR=0.47	0.27–0.81	0.006

## Discussion

Anxiety and depression worsen ACS progression and increase cardiovascular risks, while ACS symptoms and treatment stress further intensify these emotional disorders. This cycle affects all stages of ACS, from onset to recovery (24, 25). In this study, it was found that the anxiety and depression levels of patients receiving continuous home care significantly improved compared with the routine care group, resulting in a reduction in the occurrence of adverse cardiovascular events and improved quality of life of patients. The findings of this study are consistent with previous reports on psychological interventions for patients with ACS, but they include more comprehensive assessment criteria and a longer follow-up period. For example, Humphries et al. found that internet-based cognitive behavioral therapy (CBT) can alleviate depressive symptoms in patients after myocardial infarction, but this intervention lacks the multidisciplinary family care model used in this study (26). Rezaeeniya et al. also observed the beneficial effects of a continuous care model on quality of life in patients with bladder cancer, further validating the importance of long-term support (17). Unlike previous studies that focused solely on psychological indicators, this study also confirmed the improvement of intervention measures on hard cardiovascular endpoints, suggesting that mental health interventions may have direct cardiovascular protective effects.

Studies have shown that, especially for patients with cardiovascular diseases, the active implementation of scientific and reasonable nursing methods can significantly improve patients' emotional disorders, eliminate symptoms such as depression and anxiety, and also alleviate their physical symptoms such as arrhythmias and the frequency of angina attacks. The mutual influence of depression and anxiety on ACS is well-documented (27). Depression leads to platelet activation, increased blood viscosity, and platelet dysfunction, which can cause vascular damage and increase the risk of thrombus formation, thereby raising the risk of coronary events (28). In elderly patients with ACS, frailty and depression exhibit a bidirectional amplification effect. The more severe the frailty, the deeper the depression, which accelerates the decline of physical function, leading to faster progression of frailty. Our continuous home care simultaneously interrupts these two pathways of deterioration, thus potentially generating amplified benefits for elderly frail individuals (29). We also found that the patients’ depression score was significantly negatively correlated with the patients’ quality of life. Higher depression scores were associated with poorer quality of life. Effective nursing interventions that address depression and anxiety in cardiovascular patients are negatively correlated with the severity of these emotional disorders, and improving social support and utilization can alleviate depression and anxiety, thereby enhancing survival quality (30, 31). Effective nursing includes improving patients' self-management skills and treatment adherence to achieve better treatment outcomes.

In this study, it was found that compared with the routine care group, the anxiety and depression scores of patients in the continuous home care group were lower at 6, 12, and 18 months of follow-up. In addition, there was a significant difference in the quality of life of the two groups, with the continuous home care group showing superior outcomes. This may be attributed to the decreased adherence to routine care over time postdischarge. Patients may not fully recognize the benefits of a healthy lifestyle or may face difficulties in changing harmful behaviors because of a lack of the following factors: family support, timely guidance from healthcare providers, and necessary rehabilitation knowledge and skills. Continuous home care is a global nursing model developed in the past 30 years. It extends inpatient care services to home or community settings, emphasizing continuity and coordination of care plans between hospitalization and discharge. Under the guidance of the concept and model of continuous home care, nursing scholars, led by the United States, have actively explored ways to improve the nursing satisfaction, treatment adherence, and quality of life of patients with chronic diseases, and have achieved positive results (32–34). In this study, we further compared the impact of two different nursing methods on adverse cardiovascular events during the follow-up period of patients. We found that the incidence of adverse cardiovascular events was lower in the continuous home care group than in the routine care group, which improved the prognosis of patients.

Patients with ACS facing severe cardiovascular disease for the first time were selected for this study to compare the effects of various nursing techniques on their prognosis. Presently, there are no prospective studies on this topic. However, the sample size of this study is relatively small and limited to a single center, which may restrict the clinical applicability of the findings. Although we employed random sequence generation, allocation concealment, and blinded assessment of endpoint indicators to reduce the risk of bias, potential selection bias and observer bias cannot be completely ruled out. The PHQ-9 assessment window (1–8 weeks after myocardial infarction) may result in heterogeneity in baseline depression severity. Patients assessed at a later stage may show lower scores because of natural recovery or early intervention, which may dilute the effect of the intervention. In addition, this study evaluated only factors such as anxiety and depression in ACS patients. Future research could include additional factors for a more comprehensive investigation. Post-MI patients with cardiac implantable electronic devices (CIEDs) face unique psychosocial challenges (device anxiety, shock fear, and body image issues) affecting 30%–40% of recipients. Our network/telephone services could provide device-specific education and coping strategies, while community services could facilitate peer-support groups. Future studies should incorporate device therapists and measures like the Florida Patient Acceptance Survey (35). Range of motion (RM) platforms provide daily data transmission for early arrhythmia/heart failure detection but can increase anxiety without psychological support. Our nursing teams could contextualize alerts biopsychosocially. Future trials should test hybrid RM-plus-our-intervention models measuring time-to-intervention and cost-effectiveness (36, 37). The ejection fraction heterogeneity of our ACS population may obscure differential responses. Heart failure with reduced ejection fraction (HfrEF) patients may benefit more from home visits because of a higher symptom burden, while heart failure with preserved ejection fraction (HfpEF) patients may prefer network services. Future studies should prespecify ejection fraction subgroups.

## Conclusion

This prospective cohort study demonstrated that continuous home care significantly improved mental health and quality of life in patients with ACS compared with standard care. The study found that continuous home care alleviated symptoms of depression and anxiety, and the intervention group experienced improved quality of life and reduced cardiovascular adverse events. These results highlight the value of integrating structured psychosocial support into post-ACS care, consistent with the biopsychosocial model. After adjusting for confounding factors such as age, history of MI, heart failure, and medication adherence, the intervention effect did not show significant attenuation, further confirming its independent protective role.

## Data Availability

The original contributions presented in the study are included in the article/Supplementary Material, and further inquiries can be directed to the corresponding author.
